# Clinical outcomes following five-day accelerated intermittent theta burst stimulation for treatment-resistant depression in routine clinical practice: A prospective naturalistic cohort study

**DOI:** 10.1017/S1092852926101205

**Published:** 2026-10-15

**Authors:** Asmi Khanna, Adrian Espiritu, Lynn Zhu, Mervin Blair, Olivia Giovannetti, Amanda Abate, Janice Knight, Luisa Deutsch, Robyn Waxman, Amer M. Burhan

**Affiliations:** 1 Department of Human Biology, Faculty of Arts & Science, University of Toronto, Toronto, Ontario, Canada; 2 Ontario Shores Centre for Mental Health Sciences, Whitby, Ontario, Canada; 3 Lawson Health Research Institute, London, ON, Canada; 4 Department of Psychiatry, Temerty Faculty of Medicine, University of Toronto, Toronto, Ontario, Canada

**Keywords:** Accelerated transcranial magnetic stimulation, treatment-resistant depression, major depressive disorder, bipolar II disorder, depression

## Abstract

**Objectives:**

Treatment-resistant depression (TRD) is a debilitating condition occurring in major depressive disorder (MDD) and bipolar II disorder (BP-II). Intermittent theta burst stimulation (iTBS) is an established neuromodulation approach for TRD, but standard delivery requires once-daily sessions over several weeks. Accelerated iTBS (aiTBS) may reduce treatment burden by temporally compressing delivery. We evaluated the implementation, tolerability, and clinical outcomes associated with a 5-day aiTBS schedule for adults experiencing TRD in MDD and BP-II.

**Methods:**

In this single-center, open-label, uncontrolled, prospective naturalistic cohort study, adults with MDD or BP-II experiencing TRD received 30 iTBS sessions (600 pulses/session; total 18 000 pulses) over 5 days with 50-minute inter-session intervals. HAMD-17 was assessed at baseline, post-treatment, and 2-month follow-up. Secondary outcomes included Patient Health Questionnaire-9 (PHQ-9), Generalized Anxiety Disorder-7 (GAD-7), baseline Clinical Global Impression-Severity (CGI-S), post-treatment/follow-up CGI-I, response/remission rates, and adverse events. Longitudinal changes were evaluated using linear mixed-effects models.

**Results:**

Forty-six participants contributed longitudinal data (mean age 44.3 years; 54.4% male). HAMD-17 response rates were 28.3% (*n* = 13) post-treatment and 37.0% (*n* = 17) at follow-up; remission rates were 26.1% (*n* = 12) and 32.6% (*n* = 15), respectively. Improvements were also observed on PHQ-9 and GAD-7. On CGI-I, 52.2% were rated improved post-treatment and 60.9% at follow-up. The most common adverse event was transient, non-serious stimulation-site pain.

**Conclusions:**

The 5-day aiTBS schedule was logistically feasible, well-tolerated, and associated with reductions in depressive and anxiety symptom scores in a real-world TRD cohort. Randomized sham-controlled trials are needed to confirm these findings.

## Introduction

Treatment-resistant depression (TRD) represents a substantial clinical and health-system burden. Approximately one-third of individuals with depression fail to achieve adequate response to pharmacological or psychotherapeutic interventions and are classified as having TRD.^1^ TRD is associated with substantially worse clinical outcomes, including greater functional impairment, higher rates of psychiatric and medical comorbidity, increased suicidality, and diminished quality of life.^2^ Individuals with TRD also disproportionately contribute to healthcare utilization, with higher rates of outpatient visits, hospitalizations, and overall system costs compared with those who respond to first-line treatment.^3^ Although historically examined within major depressive disorder (MDD), the construct of treatment resistance is also applied in bipolar disorders to denote refractoriness to multiple therapeutic interventions.^4^

Contemporary neurobiological accounts conceptualize depression as a disorder of distributed brain networks, including network-level dysconnectivity and impaired synaptic plasticity.^5^ ^–^^8^ These mechanisms provide a rationale for circuit-targeted neuromodulatory interventions.

The Canadian Network for Mood and Anxiety Treatments (CANMAT) 2023 guidelines recommend, following inadequate response to first-line pharmacotherapy or psychotherapy, optimization strategies, pharmacological augmentation, and consideration of neuromodulation approaches such as repetitive transcranial magnetic stimulation (rTMS) for TRD.^9^ rTMS is a non-invasive neuromodulatory intervention that delivers rapidly alternating magnetic fields to depolarize cortical neurons and modulate targeted cortical regions, most commonly the left dorsolateral prefrontal cortex (L-DLPFC).^10^ By modulating cortical excitability and synaptic efficacy, rTMS influences distributed fronto-limbic and fronto-striatal networks implicated in affect regulation, cognitive control, and reward processing.^11^ ^,^^12^ Regulatory approval for rTMS in TRD was granted by Health Canada in 2002 and by the United States Food and Drug Administration (US FDA) in 2008.^13^ ^,^^14^

Multiple standard rTMS protocols were endorsed by the CANMAT 2023 guidelines as first-line neuromodulation strategies for MDD, including intermittent theta burst stimulation (iTBS), high-frequency stimulation (10 Hz) of the L-DLPFC, and low-frequency stimulation (1 Hz) to the right-DLPFC. These protocols are conventionally administered once daily over 4–6 weeks (ie, 20–30 daily sessions in total).^9^ Although efficacious, the temporal demands of standard rTMS courses impose substantial real-world barriers. Daily clinic attendance over several consecutive weeks necessitates repeated travel, time away from employment or caregiving responsibilities, and sustained logistical coordination.^13^ Such demands may disrupt occupational and family functioning, and may limit scalability by constraining clinic throughput.^13^

Since individual iTBS sessions are brief (~3 minutes), this protocol permits delivery of multiple stimulation sessions within a single day.^15^ Accelerated TMS protocols were developed to condense the treatment course by administering several sessions per day across a limited number of consecutive treatment days.^16^ Conceptually, accelerated iTBS (aiTBS) preserves the cumulative pulse exposure of conventional iTBS while altering the temporal distribution of treatment, potentially harnessing cumulative plasticity effects within a compressed timeframe. Mechanistically, repeated iTBS sessions delivered within days may engage both rapid within-session plasticity and slower consolidation processes between sessions, while inter-session intervals may modulate whether successive bouts produce additive potentiation, homeostatic downregulation, or metaplastic priming. In addition to addressing patient burden, this approach may enhance accessibility and system efficiency.

aiTBS is currently regarded as a second-line TMS protocol for TRD in MDD, and existing evidence remains heterogenous, with substantial variability in stimulation parameters and reported outcomes.^9^ Preliminary studies delivering up to 36 theta burst sessions over 5 days have demonstrated promising, though variable, response and remission rates.^17^ However, real-world data describing the implementation, tolerability, and short-term clinical outcomes of temporally compressed accelerated protocols delivered in routine clinical practice remain limited.

In this naturalistic, single-center observational study, we evaluated the implementation, tolerability (ie, occurrence of adverse events [AE]), and preliminary clinical outcomes of aiTBS among adults experiencing a treatment-resistant major depressive episode (MDE) in the context of MDD or BP-II, with follow-up extending approximately 2 months after treatment completion.

## Methods

### Study design

We conducted a single-center, open-label, uncontrolled, prospective, naturalistic cohort study to evaluate the implementation, tolerability, and preliminary clinical outcomes associated with aiTBS in adults experiencing an MDE in the context of MDD or BP-II.

Given the absence of randomization or a sham comparator, the study characterized treatment implementation and longitudinal symptom trajectories following condensed aiTBS protocol (ie, up to 30 sessions over 5 days), rather than to establish causal treatment efficacy.

#### Ethical approval

The Ontario Shores Research Ethics Board approved the study in accordance with OS REB #21–008-B and JREB #23–007-B).

### Setting

The study was conducted at the specialized brain stimulation clinic of Ontario Shores Centre for Mental Health Sciences, a public tertiary psychiatric teaching hospital located in Ontario, Canada.

### Participants

Participants were consecutively recruited from individuals referred for clinical rTMS consultation at the Brain Stimulation Clinic. Eligibility was determined through routine clinical assessment by a brain stimulation-trained and credentialed psychiatrist.

TRD was operationalized as failure to achieve adequate clinical response following at least 2 antidepressant trials of adequate dose and duration, as documented in the medical record.

Participants met inclusion criteria if they satisfied all of the following: (a) age ≥ 18 years; (b) presence of a current non-psychotic MDE in the context of MDD or BP-II; (c) fulfillment of TRD criteria as defined above; (d) clearance on a standardized rTMS safety screening assessment; and (e) capacity and willingness to provide informed consent for treatment.

Patients were excluded if they met any of the following criteria: (a) diagnosis of bipolar I disorder; (b) lifetime diagnosis of a schizophrenia spectrum or other primary psychotic disorder; (c) recent substance use disorder; (d) unstable or clinically significant medical or neurological illness; (e) pregnancy or possible pregnancy; (f) presence of intracranial implants or ferromagnetic material near the head; or (g) concurrent use of benzodiazepines exceeding lorazepam 2 mg/day or equivalent, or use of anticonvulsant medication.

Psychotropic medication regimens were required to remain stable during the 5-day acute treatment period. Concomitant benzodiazepine use was permitted only when prescribed on an as-needed basis and not exceeding lorazepam 2 mg per 24 hours or equivalent. Patients were instructed not to use benzodiazepines after 5 PM on the evening preceding daily rTMS treatment. Non-benzodiazepine anxiolytic use was permitted when clinically indicated and documented in the medical record. Changes during follow-up were permitted and documented in clinical records, but were not protocolized.

Participants with BP-II were included because recruitment occurred through a routine tertiary brain stimulation service in which clinical eligibility for aiTBS was based on the presence of a treatment-resistant depressive episode rather than diagnosis alone. Given the small number of BP-II participants, diagnostic subgroup analyses were not performed. However, a sensitivity analysis excluding BP-II participants was conducted to determine whether their inclusion materially influenced the primary depressive symptom findings.

Participants were followed from baseline assessment through completion of the aiTBS treatment course and reassessed at post-treatment and approximately 8 weeks (±2 weeks) following treatment as part of routine clinical outcome monitoring.

### Outcomes and variables

Baseline demographic and clinical variables were recorded at treatment initiation, with clinical outcomes reassessed at the post-treatment and follow-up period.

#### Primary outcome

The prespecified primary clinical outcome was longitudinal change in depressive symptom severity from baseline to immediate post-treatment, measured continuously using the 17-item Hamilton Depression Rating Scale (HAMD-17). Change in HAMD-17 from baseline to approximately 2-month follow-up was evaluated as a key secondary durability outcome. The HAMD-17 was administered by credentialed brain stimulation psychiatrists at baseline, post-treatment, and follow-up.

HAMD-17 response was operationalized a priori as a ≥ 50% reduction in scores relative to baseline. HAMD-17 remission was defined as HAMD-17 ≤ 7. Categorical response and remission rates were calculated descriptively at post-treatment and follow-up.

#### Secondary outcomes

Secondary outcomes included longitudinal changes in self-rated depressive symptoms, anxiety symptoms, clinician-rated global clinical change, categorical symptom response, remission rates, and tolerability.

Continuous symptom severity was assessed using validated clinician- and patient-reported measures at baseline, post-treatment, and follow-up: (a) depressive symptom severity measured using the Patient Health Questionnaire-9 (PHQ-9); (b) anxiety symptom severity was measured using the Generalized Anxiety Disorder-7 (GAD-7); (c) baseline clinician-rated global illness severity assessed using the Clinical Global Impression-Severity (CGI-S) scale. Post-treatment and follow-up clinician-rated global clinical change were assessed using the Clinician Global Impression-Improvement (CGI-I) scale. Because CGI-S and CGI-I assess different constructs, CGI was not analyzed as a continuous longitudinal outcome across baseline, post-treatment, and follow-up. CGI-I was summarized descriptively at post-treatment and follow-up and was not used to define remission.

Clinically meaningful symptom response was defined as reduction relative to baseline according to the following thresholds: HAMD-17 ≥ 50% reduction, PHQ-9 ≥ 6-point reduction, and GAD-7 ≥ 4-point reduction. Remission was defined using symptom severity thresholds for HAMD-17, PHQ-9, and GAD-7, but not CGI-I.

Tolerability was assessed by the occurrence of AE recorded at each aiTBS treatment session.

### Accelerated intermittent theta burst stimulation protocol

aiTBS sessions were administered by trained rTMS technicians under physician supervision. Stimulation was delivered to the L-DLPFC 6 times daily over 5 consecutive days, for a maximum of 30 treatment sessions per participant.

Inter-session intervals were at least 50 minutes. Each iTBS session lasted approximately 3 minutes.

Stimulation was delivered using a Magpro X100 stimulator (Magventure, Denmark) equipped with a B70 fluid-cooled figure-of-eight coil. Target localization of the L-DLPFC was performed using the Beam F3 method, consistent with standard clinical practice in non-image-guided rTMS settings. Resting motor threshold (RMT) was determined using standard clinical procedures. The intended target stimulation intensity was 120% of individual RMT, consistent with standard antidepressant iTBS protocols.^18^ A tolerability-based titration range from 90% to 120% RMT was permitted during treatment initiation, such that stimulation could be temporarily adjusted according to individual tolerability and titrated toward the target intensity as clinically tolerated.^18^ Because participant-level delivered stimulation intensity data were not available with sufficient granularity, we were unable to determine the proportion of participants treated at each intensity level or to examine associations between delivered intensity, clinical outcomes, and adverse events.

**Figure 1.**
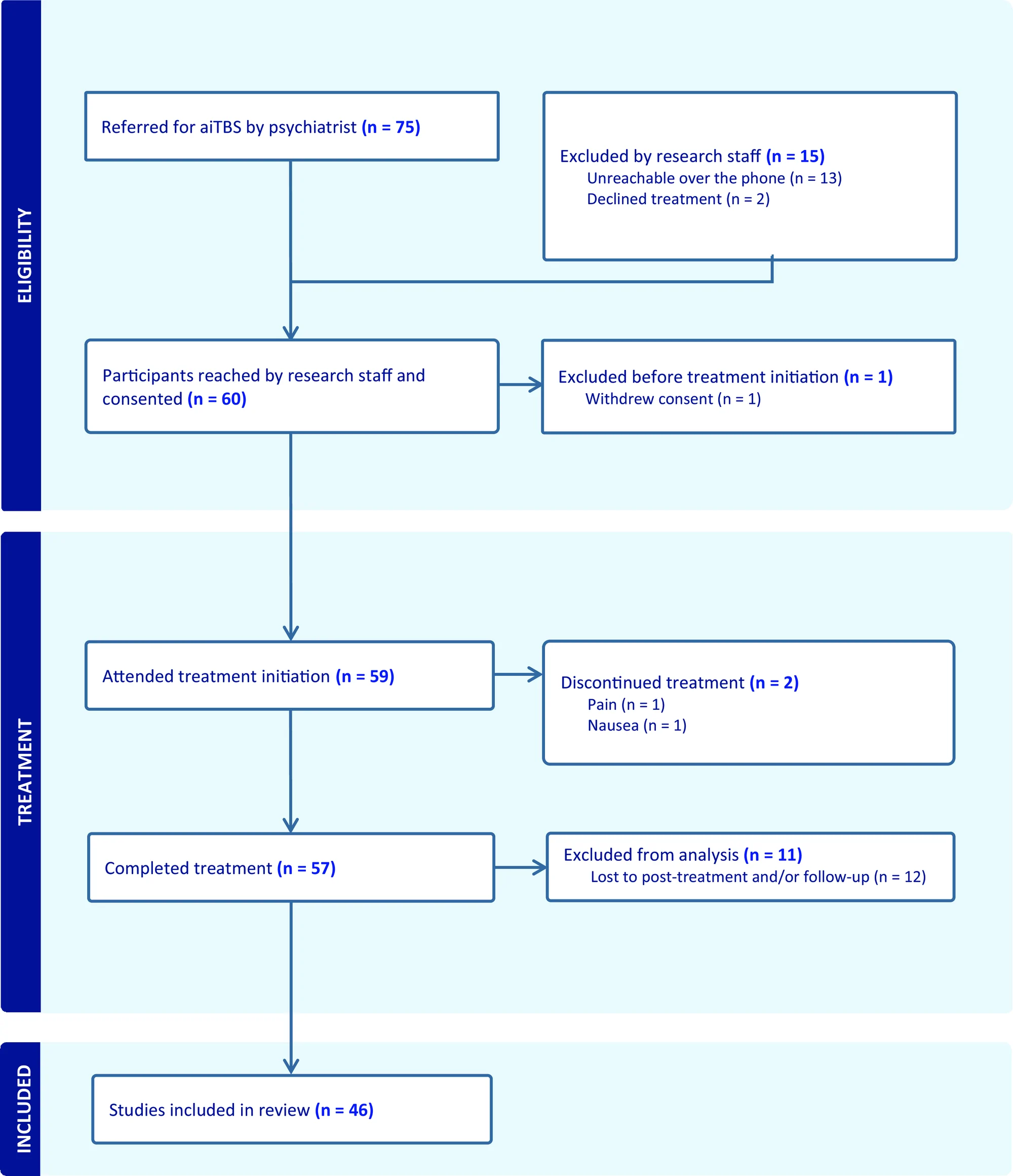
Flow of participants from psychiatric referral to final analytic sample in a prospective naturalistic accelerated iTBS study. Figure 1. long description.

Each iTBS session consisted of 600 pulses delivered as bursts of 3 pulses at 50 Hz, repeated at 5 Hz. Stimulation was delivered in 2-second trains followed by 8-second inter-train intervals, for a total of 20 trains over approximately 3 minutes per session (Figure 1). Across the full treatment course, participants received up to 30 sessions corresponding to 18 000 total pulses. This cumulative pulse exposure is consistent with standard once-daily iTBS protocols administered over 4-to-6 weeks, but was delivered over a temporally compressed schedule.

### Bias and confounding considerations

As a naturalistic, open-label study, several potential sources of bias and confounds were considered.

Selection bias may have arisen because participants were recruited from a tertiary referral center and received treatment within a specialized brain stimulation service. To mitigate this, consecutive recruitment of clinically eligible patients referred for routine rTMS consultation was employed.

Performance and expectancy biases are inherent to open-label neuromodulation studies. To reduce measurement bias, validated clinician-rated and self-reported scales were administered at standardized clinical time points as part of routine outcome monitoring. Clinician-rated assessments were conducted by trained clinical staff using standard rating procedures.

Confounding by concurrent treatments is possible, particularly during the follow-up period. Medication regimens were required to be stable during the acute 5-day aiTBS treatment phase; however, changes during follow-up were not protocolized and may have influenced longer-term outcomes.

Concomitant benzodiazepine or anxiolytic exposure represents an additional potential confounder. Patients receiving benzodiazepines exceeding lorazepam 2 mg/day or equivalent, or anticonvulsant medications, were excluded. However, benzodiazepine use within the permitted dose range and/or other anxiolytic medication use was recorded from clinical records at baseline. Permitted benzodiazepines were prescribed on an as-needed basis with instructions not to exceed lorazepam 2 mg per 24 hours or equivalent and not to use benzodiazepines after 5 PM on the evening preceding daily rTMS treatment. Actual daily consumption, dose timing, duration, frequency of as-needed use, and changes in anxiolytic exposure during follow-up were not available with sufficient granularity to support reliable differential-response analyses. Delivered stimulation intensity may also have contributed to clinical variability; however, participant-level delivered RMT intensity data were not available with sufficient granularity to support intensity-response or intensity-tolerability analyses.

Regression to the mean and spontaneous symptom fluctuation cannot be excluded in the absence of a control group. Findings should therefore be interpreted as preliminary clinical outcome signals rather than definitive causal effects.

### Sample size considerations

A formal a priori sample size calculation was not performed, as the study was designed to generate preliminary implementation, tolerability, and clinical outcome estimates rather than to test a prespecified efficacy hypothesis.

An anticipated sample size of approximately 50 participants was determined pragmatically based on expected referral volume, eligibility rates, and the clinic capacity. The final analytic sample comprised 46 participants.

This study was not powered to detect between-group differences or to perform multivariable predictor modelling.

### Statistical analysis

Descriptive statistics were used to summarize baseline demographic and clinical characteristics. Continuous variables are reported as means and standard deviations; categorical variables are reported as counts and percentages. Annotated R scripts used to perform all analyses are provided in the Supplementary Materials.

Longitudinal changes in HAMD-17, PHQ-9, and GAD-7 scores across baseline, post-treatment, and follow-up were analyzed using linear mixed-effects models. Models were fitted using maximum likelihood estimation for model comparison and restricted maximum likelihood for final parameter estimation. Time was modeled as a categorical fixed effect, and participant-level random intercepts were included to account for within-subject correlation of repeated measures. Degrees of freedom were estimated using the Satterthwaite approximation. For the primary outcome, the principal contrast of interest was baseline to immediate post-treatment change in HAMD-17. The baseline to approximately 2-month follow-up HAMD-17 contrast was interpreted as a secondary durability analysis. Longitudinal changes in PHQ-9 and GAD-7 were interpreted as secondary continuous outcome analyses. CGI-S was summarized as a baseline measure of clinician-rated global illness severity, while CGI-I was summarized descriptively at post-treatment and follow-up.

When the overall time effect was significant, pairwise comparisons between time points were conducted using estimated marginal means with Bonferroni adjustment (*p* < .017). Standardized effect sizes (Cohen’s d) with 95% confidence intervals (CI) were calculated based on model-derived estimates. Cohen’s d was specifically calculated using model-estimated marginal means and pooled baseline standard deviation.

To assess whether inclusion of participants with BP-II influenced the primary findings, a sensitivity analysis was conducted by repeating the primary HAMD-17 mixed-effects model after excluding the 3 BP-II participants.

Categorical response and remission outcomes for HAMD-17, PHQ-9, and GAD-7 were summarized descriptively and were not subjected to inferential hypothesis testing. CGI-I was summarized descriptively as a measure of clinician-rated global clinical change at post-treatment and follow-up, and was not used to define response or remission.

All tests were two-sided with *α* = 0.05. Analyses were performed using RStudio version 2026.01.1 + 403 with the lme4, lmerTest, and emmeans packages.

## Results

### Participant demographics and feasibility of aiTBS treatment

A total of 46 participants were included in the analytic sample (Table 1). The mean age was 44.3 years (range 21–77), and 54.4% (*n* = 25) were male. The majority were diagnosed with MDD (93.5%, *n* = 43), with a small proportion diagnosed with Bipolar II Disorder (6.5%, *n* = 3).

**Table 1.** Baseline Demographic and Clinical Characteristics of Participants (*n* = 46) Table 1. long description.

Variable	Value
Age, mean (range)	44.33 (21–77)
Sex, *n* (%)	
Male	25 (54.4)
Female	21 (45.7)
Diagnosis, *n* (%)	
Major depressive disorder	43 (93.5)
Bipolar Disorder-II	3 (6.5)
Prior and concurrent treatments, *n* (%)	
Atypical antipsychotics	25 (54)
Benzodiazepines or other anxiolytics	28 (61)
Psychotherapy	33 (71)
Electroconvulsive therapy	11 (24)
Baseline symptom severity, *mean* (SD)	
CGI–S	4.35 (0.67)
HAMD–17	17.59 (5.69)
GAD–7	10.76 (5.50)
PHQ–9	18.63 (5.16)

Abbreviations: CGI–S, Clinical Global Impression–Severity scale; GAD–7, General Anxiety Disorder scale–7; HAMD–17, Hamilton Depression Rating Scale–17; PHQ–9, Patient Health Questionnaire–9.

Participants demonstrated substantial prior treatment exposure consistent with TRD. All participants with available treatment-history data had failed at least 2 antidepressant trials of adequate dose and duration. Adjunctive atypical antipsychotics were prescribed in 25 participants (54.3%). Although patients receiving benzodiazepines exceeding lorazepam 2 mg/day or equivalent, or anticonvulsant medications, were excluded, 28 participants (60.9%) had permitted benzodiazepines and/or other anxiolytic exposure during the aiTBS treatment course. A majority had previously engaged in structured psychotherapy, including cognitive behavioral therapy or dialectical behavioral therapy (71.7%, *n* = 33), and 23.9% (*n* = 11) had undergone electroconvulsive therapy.

At baseline, depressive symptom severity was moderate to severe (HAMD-17 mean = 17.6, SD = 5.7; PHQ-9 mean = 18.6, SD = 5.2). Mean baseline GAD-7 score was 10.8 (SD = 5.5), indicating clinically significant anxiety symptoms. Mean baseline CGI-S score was 4.35 (SD = 0.67), consistent with marked illness severity.

Baseline HAMD-17 scores ranged from 4 to 34. One participant (2.2%) had a baseline HAMD-17 score in the remission/minimal-symptom range (≤7), 22 participants (47.8%) had mild symptoms (HAMD-17 8–16), 14 participants (30.4%) had moderate symptoms (HAMD-17 17–23), and 9 participants (19.6%) had severe symptoms (HAMD-17 ≥ 24). Eligibility for aiTBS was determined through routine clinical assessment of a treatment-resistant MDE rather than by a prespecified minimum HAMD-17 threshold.

**Figure 2.**
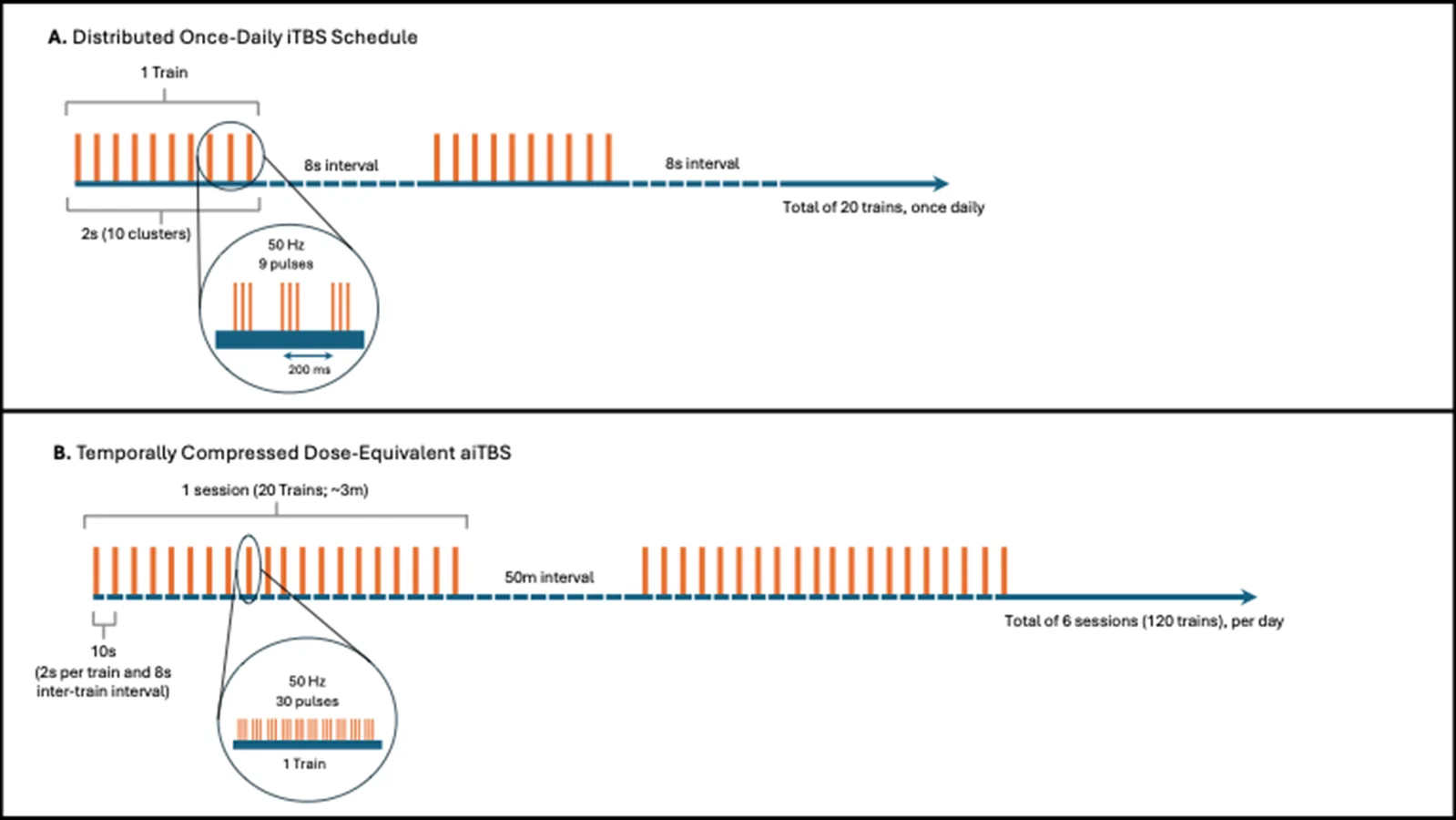
Temporal architecture of conventional once-daily iTBS versus 5-day aiTBS. Schematic illustration of (a) conventional once-daily intermittent theta burst stimulation (iTBS) and (b) accelerated iTBS (aiTBS). In both protocols, each session consists of 20 trains delivered over approximately 3 minutes (600 pulses), with identical burst frequency and intra-train structure. The distinction lies in session spacing. Conventional iTBS distributes 1 session per day across 4–6 weeks, whereas the accelerated protocol delivers 6 identical sessions per day separated by ~50-minute inter-session intervals, completing treatment over 5 consecutive days. Thus, cumulative pulse exposure (18 000 pulses) is preserved, while the temporal distribution of stimulation is compressed. The figure highlights that the accelerated approach modifies treatment chronology rather than per-session dose or stimulation parameters. Figure 2. long description.

Of 59 participants who initiated treatment, 46 contributed longitudinal outcome data and were included in the analytic sample (Figure 2). The mixed-effects modeling approach incorporated all available observations for HAMD-17, PHQ-9, and GAD-7, allowing inclusion of participants with incomplete follow-up assessments (*n* = 7).

### Longitudinal clinical outcomes

#### Primary outcome

Linear mixed-effects modeling demonstrated a significant effect of time on HAMD-17 scores.

For the prespecified primary endpoint, HAMD-17 scores decreased significantly from baseline to immediate post-treatment (mean difference = −5.26, SE = 1.11*, t* = −4.72, *p* < .001). In the secondary durability analysis, HAMD-17 scores remained significantly reduced at approximately 2-month follow-up relative to baseline (mean difference = −5.31, SE = 1.19, *t* = −4.46, *p* < .001).

There was no significant difference between post-treatment and follow-up scores (p = 1.00), indicating maintenance of treatment gains.

The standardized within-subject effect size for baseline to post-treatment change was large (Cohen’s *d* = 1.00, 95% CI [0.56, 1.45]). A similarly large observed within-subject change was found at follow-up (*d* = 1.01, 95% CI [0.55, 1.48]).

In a sensitivity analysis excluding the 3 participants with BP-II, the primary HAMD-17 findings remained materially unchanged. Among participants with MDD only (*n* = 43), there was a significant effect of time on HAMD-17 scores, F(2, 74.22) = 13.26, *p* < .001. HAMD-17 scores were significantly reduced from baseline to post-treatment (mean difference = −5.17, SE = 1.17, *t* = −4.42, Bonferroni-adjusted *p* < .001) and from baseline to follow-up (mean difference = −5.37, SE = 1.23, *t* = −4.36, Bonferroni-adjusted *p* < .001). There was no significant difference between post-treatment and follow-up scores (mean difference = −0.19, SE = 1.26, *t* = −0.15, Bonferroni-adjusted *p* = 1.00). These findings indicate that the primary depressive symptom trajectory was not driven by the small BP-II subgroup.

#### Secondary outcomes

PHQ-9 scores demonstrated a significant effect of time. Compared with baseline, mean PHQ-9 scores were significantly reduced at post-treatment (mean difference = −4.87, SE = 0.87, *t* = −5.59, *p* < .001) and at follow-up (mean difference = −5.43, SE = 0.96, *t* = −5.68, *p* < .001). No significant difference was observed between post-treatment and follow-up scores (*p* = 1.00), indicating maintenance of observed symptom reductions. The magnitude of observed within-subject change was large, with effect sizes of *d* = 1.17 (95% CI [0.73, 1.61]) from baseline to post-treatment and *d* = 1.30 (95% CI [0.82, 1.78]) from baseline to follow-up.

Anxiety symptoms measured by the GAD-7 also showed a significant effect of time. Relative to baseline, GAD-7 scores were significantly lower at post-treatment (mean difference = −2.46, SE = 0.67, *t* = −3.64, *p* = .0015) and at 2-month follow-up (mean difference = −2.52, SE = 0.75, *t* = −3.35, *p* = .0037). No significant difference was observed between post-treatment and follow-up (*p* = 1.00), indicating maintenance of observed reductions in anxiety symptoms. The corresponding effect sizes were moderate to large, with *d* = 0.76 (95% CI [0.33, 1.19]) at post-treatment and *d* = 0.78 (95% CI [0.30, 1.25]) at follow-up.

Baseline clinician-rated global illness severity was assessed using CGI-S, with a mean score of 4.35 (SD = 0.67), consistent with marked illness severity. Post-treatment and follow-up clinician-rated global clinical change were assessed using CGI-I and summarized descriptively. Because CGI-S and CGI-I assess different constructs, CGI scores were not analyzed as a continuous longitudinal outcome across baseline, post-treatment, and follow-up.

### Categorical response, remission, and CGI-I improvement outcomes

**Figure 3.**
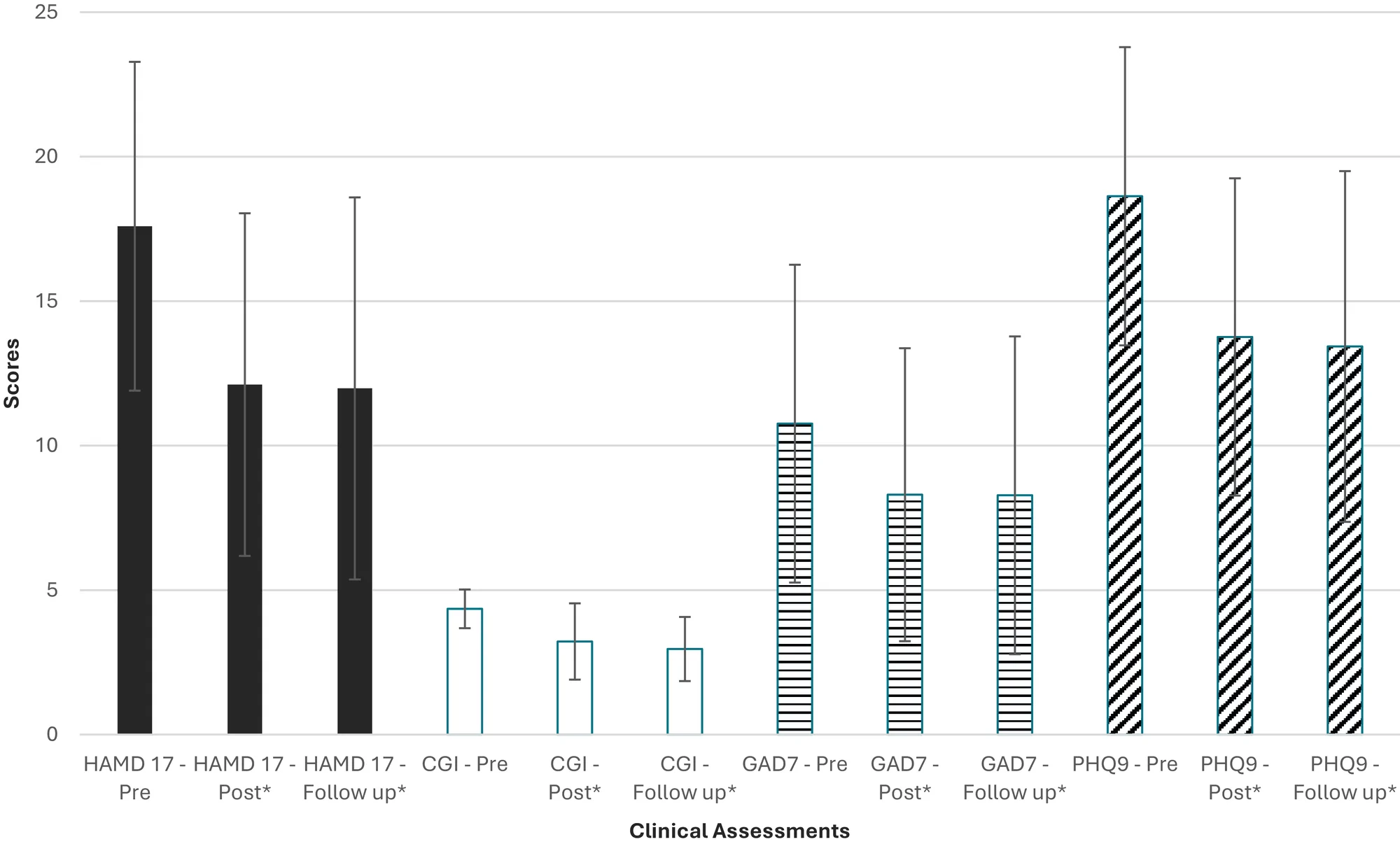
Average clinical assessment scores taken at baseline, post-treatment, and at 2-month follow-up (statistically significant improvement in scores relative to baseline is labelled with (*)) (Standard deviation bars displayed). Figure 3. long description.

Categorical response and remission outcomes were analyzed descriptively for HAMD-17, PHQ-9, and GAD-7 (Table 2 **;** Figure 3). CGI-I improvement ratings were summarized descriptively at post-treatment and follow-up.

**Table 2.** Participant Scores on the HAMD-17, PHQ-9, GAD-7, and CGI at Baseline, Post-Treatment, and at 2-Month Follow-Up After the Accelerated Intermittent Theta Burst Stimulation Protocol Table 2. long description.

	Mean scores ± SD (Range), *n* = 46			% of participants meeting symptom reduction criteria, relative to baseline (*n* = 46)		% of participants meeting remission criteria^h^ (*n* = 46)	
	Baseline	Post-treatment	2-mo follow-up	Post-treatment	2-mo follow-up	Post-treatment	2-mo follow-up
HAMD-17	17.6 ± 5.7 (4–34)	12.1 ± 5.9 (3–25)^a^	12.0 ± 6.6 (2–25)^a^	Improved and meeting clinically meaningful criteria^b^: 28.3% (*n* = 13) Improved, did not meet criteria^c^: 50.0% (*n* = 23) No change^e^: 6.5% (*n* = 3) Worsened^f^: 15.2% (*n* = 7)	Improved and meeting clinically meaningful criteria^b^: 37.0% (*n* = 17) Improved, did not meet criteria^c^: 41.3% (*n* = 19) No change^e^: 6.5% (*n* = 3) Worsened^f^: 15.2% (*n* = 7)	26.1% (*n* = 12)	32.6% (*n* = 15)
PHQ-9	18.6 ± 5.2 (4–29)	13.8 ± 5.5 (4–24)^a^	13.4 ± 6.1 (2–25)^a^	Improved^d^: 80.4% (*n* = 37) No change^e^: 4.3% (*n* = 2) Worsened^f^: 15.2% (*n* = 7)	Improved^d^: 78.3% (*n* = 36) No change^e^: 6.5% (*n* = 3) Worsened^f^: 15.2% *(n* = 7)	2.2% (*n* = 1)	6.5% (*n* = 3)
GAD-7	10.8 ± 5.5 (0–20)	8.3 ± 5.1 (1–20)^a^	8.3 ± 5.5 (0–20)^a^	Improved^d^: 60.9% (*n* = 28) No change^e^: 10.9% (*n* = 5) Worsened^f^: 28.3% (*n* = 13)	Improved^d^: 65.2% (*n* = 30) No change^e^: 6.5% (*n* = 3) Worsened^f^: 28.3% (*n* = 13)	28.3% (*n* = 13)	32.6% (*n* = 15)
CGI-I	4.3 ± 0.67 (2–5)	3.2 ± 1.3 (1–6)^a^	3.0 ± 1.1 (1–5)^a^	Improved^a^: 52.2% (*n* = 24) No change^g^: 37.0% (*n* = 17) Worsened^g^: 10.9% (*n* = 5)	Improved^g^: 60.9% (*n* = 28) No change^g^: 32.6% (*n* = 15) Worsened^g^: 6.5% (*n* = 3)	8.7% (*n* = 4)	6.5% (*n* = 3)

*Note:* Higher scores denote worse symptoms across all 4 scales.

Abbreviations: CGI, Clinical Global Impression-Improvement; GAD-7, General Anxiety Disorder scale-7; HAMD-17, Hamilton Depression Rating Scale-17; PHQ-9, Patient Health Questionnaire-9; SD, Standard deviation.

a Statistically significant differences are marked with * compared to baseline data (Bonferroni corrected *p* < 0.017);

b “Improved and meeting clinically meaningful criteria”: HAMD-17 ≥ 50% reduction compared to baseline);

c Improved, did not meet criteria: any improvement defined as any reduction in HAMD-17 but did not reach the ≥50% reduction compared to baseline);

d “Improved”: any improvement defined as any reduction in PHQ-9 or GAD-7 scores compared to baseline;

e “No change”: defined as no change in scores in HAMD-17, PHQ-9 or GAD-7 compared to baseline;

f “Worsened”: any worsening defined as any increase in HAMD-17, PHQ-9 or GAD-7 scores compared to baseline;

g CGI-I: “Improved” (ie, score of 1, 2, or 3); “No change” (ie, score of 4); “Worsened” (ie, score of 5, 6, or 7);

h Remission criteria defined as HAMD-17 ≤7, PHQ-9 < 5, and GAD-7 ≤ 4. CGI-I was used for descriptive improvement and not to define remission.

Based on HAMD-17 criteria, 28.3% of participants (*n* = 13) met response criteria at post-treatment, increasing to 37.0% (*n* = 17) at follow-up. HAMD-17 remission was achieved by 26.1% (*n* = 12) at post-treatment and 32.6% (*n* = 15) at follow-up.

Any reduction in PHQ-9 was observed in 80.4% of participants (*n* = 37) at post-treatment and 78.3% (*n* = 36) at follow-up. PHQ-9 remission was observed in 2.2% (*n* = 1) at post-treatment and 6.5% (*n* = 3) at follow-up.

Any reduction in GAD-7 was observed in 60.9% of participants (*n* = 28) at post-treatment and 65.2% (*n* = 30) at follow-up. GAD-7 remission was observed in 28.3% of participants (*n* = 13) at post-treatment and 32.6% (*n* = 15) at follow-up.

On CGI-I, 52.2% of participants (*n* = 24) were rated as improved at post-treatment, increasing to 60.9% (*n* = 28) at follow-up. A CGI-I rating of 1, corresponding to “very much improved,” was observed in 7–9% of participants. CGI-I was not used to define remission.

### Adverse events

The most frequently reported adverse event was pain at the stimulation site. Across 30 treatment sessions, mean reported pain was moderate (mean = 3.91 on a 1–10 scale; mode = 3; range: 1–8).

On Day 1 of treatment, 24–35% participants reported pain >5; by Day 5 this proportion decreased to 22–24%. No progressive worsening of pain severity was observed across treatment days.

Other AE including headaches, eye discomfort, nausea, and fatigue were infrequently reported (< 3% of participants).

Two participants discontinued treatment: 1 due to pain and 1 due to nausea. No serious AE were noted throughout the treatment course. For participants who completed treatment, all reported adverse symptoms were transient.

## Discussion

### Temporally compressed iTBS was associated with sustained dimensional symptom improvement in a treatment-resistant cohort

The present study evaluated clinical outcomes following compression of a standard cumulative iTBS pulse exposure into a 5-day schedule in a tertiary TRD population. Longitudinal modeling demonstrated statistically significant reductions in clinician-rated depressive symptom severity that were sustained at 2-month follow-up. Although categorical remission rates were modest immediately post-treatment, continuous symptom reductions were substantial, and the magnitude of observed within-subject change was large.

Immediately post-treatment, approximately 1 in 4 participants met HAMD-17 response criteria; this increased to more than 1 in 3 patients at 2-month follow-up. This delayed increase in response suggests that observed clinical improvement may continue beyond the acute stimulation period.

A considerable proportion of patients also demonstrated reductions in PHQ-9 and GAD-7 scores at post-treatment and 2-month follow-up. Clinician-rated global clinical change, assessed using CGI-I after treatment, similarly indicated that over half of participants were rated as improved at post-treatment, increasing further at follow-up. Because baseline clinician-rated global severity was assessed using CGI-S whereas post-treatment and follow-up clinical change were assessed using CGI-I, CGI scores were interpreted descriptively rather than as a continuous longitudinal outcome. The most common AE was pain at the stimulation site, which was typically moderate and transient. Taken together, these findings support the clinical relevance of evaluating temporally compressed iTBS schedules in routine practice. Rather than establishing the feasibility of aiTBS de novo, the present study contributes prospective real-world implementation data for a pragmatic 5-day schedule delivered in a tertiary brain stimulation clinic.

### Relationship to conventional iTBS and accelerated TBS literature

A recent review identified 16 studies of accelerated TBS in MDD/TRD populations and 4 studies in bipolar disorder or mixed MDD samples.^16^ Across included studies, mean response and remission rates were 52.2% and 38.2%, respectively, while the THREE-D trial of conventional iTBS reported response and remission rates of 49% and 32% at treatment completion.^16^ ^,^^19^ In the present cohort, HAMD-17 response and remission rates were 28.3% and 26.1% immediately post-treatment, and 37.0% and 32.6% at approximately 2-month follow-up, respectively. Because the THREE-D outcomes were assessed at treatment completion whereas our highest remission estimate was observed at follow-up, this comparison should be interpreted only as a contextual benchmark rather than a direct temporal comparison. With this caveat, the present cohort showed lower immediate post-treatment response and remission rates than THREE-D, with follow-up remission numerically similar to the THREE-D treatment-completion estimate.

These findings should therefore not be interpreted as evidence that temporal compression preserves the full antidepressant signal of conventional or higher-intensity accelerated protocols. Rather, they suggest that a 5-day, 6-session-per-day schedule was associated with measurable clinical improvement while substantially reducing the time required to complete treatment. The clinical question raised by this pragmatic schedule is whether reduced treatment burden and improved deliverability may justify a potentially more modest clinical effect, a question that requires direct testing in randomized comparative trials.

### Lower remission rates relative to SNT may reflect differences in treatment-intensity, cumulative pulse exposure, and targeting strategy

*Stanford Neuromodulation Therapy (SNT)* administers 10 iTBS sessions per day with 50-minute inter-stimulation intervals, delivering 90 000 total pulses across 50 sessions.^20^ In the open-label SNT trial, 19 of 21 TRD patients (90.5%) achieved remission (ie, defined as a Montgomery-Åsberg Depression Rating Scale (MÅDRS) score of <11).^20^ ^,^^21^ In the subsequent double-blind randomized controlled trial, remission occurred in 11 of 14 participants (78.6%) receiving active SNT compared with 2 of 15 (13.3%) in the sham stimulation.^20^ These remission rates are substantially higher than those observed in the present cohort. Similarly, in a smaller prospective study by Luehr et al.,^17^ which delivered 36 aiTBS sessions over 5 days, MÅDRS scores decreased significantly over 4 weeks (*p* < .0001), with 70% and 55% of participants meeting response and remission criteria.

Several protocol-level differences may account for the more modest response and remission rates observed in our cohort. First, SNT employed individualized functional connectivity MRI-guided targeting of the L-DLPFC based on its anticorrelation with the subgenual anterior cingulate cortex, a strategy intended to maximize engagement of depression-relevant network circuitry.^20^ In contrast, our study utilized the Beam F3 scalp-based localization method, which is consistent with routine clinical practice but may introduce greater inter-individual variability in target engagement. Second, our protocol delivered 30 total sessions and 18 000 total pulses, compared with 50 sessions and 90 000 total pulses in SNT.19,20 Therefore, the lower remission rates observed in our cohort may reflect not only differences in targeting precision, but also the lower total number of sessions and cumulative pulse exposure.

This interpretation is supported by the broader accelerated rTMS literature. Accelerated rTMS protocols vary substantially in stimulation frequency, total session number, pulses per session, inter-session interval, and treatment duration.^15^ ^,^^18^ A prior review evaluated 63 accelerated rTMS trials that utilized between 9 and 104 total sessions, with 2–10 treatment sessions administered daily over 2–30 treatment days.^18^ Findings from this review suggest that higher numbers of daily sessions, total sessions, and total pulses correlate positively with the response rate. Conversely, a recent retrospective study by Kerkel et al.^22^ (*n* = 215) demonstrated that increasing iTBS pulses per session from 600 to 1200 or 1800 was not associated with greater antidepressant symptom reduction.^23^ Together, these findings suggest that total session number and cumulative pulse exposure may be more relevant to clinical outcomes than pulses per session alone.

The choice of 30-session schedule should therefore be interpreted as a deliberate trade-off between treatment intensity, routine clinical deliverability, and potential clinical effect, rather than as an attempt to maximize stimulation exposure. Recent meta-regression evidence by Pettorruso et al. found that, across accelerated high-frequency rTMS and aiTBS studies in depression, greater total pulse exposure and a higher total number of sessions were associated with favorable antidepressant response, whereas pulses per session appeared less influential than the total number of sessions delivered.^24^ Inter-session intervals of at least 50 minutes were also associated with more favorable outcomes.^24^ Our protocol incorporated this spacing principle by using 50-minute inter-session intervals, but retained 30 sessions and 18 000 total pulses to mirror the cumulative exposure of standard iTBS while compressing delivery into 5 days. This design contrasts with SNT, which combines 50 sessions, 90 000 total pulses, and individualized connectivity-guided targeting.^19^ ^,^^20^ Accordingly, the present study does not establish that a lower-intensity accelerated schedule is biologically or clinically equivalent to SNT. Rather, it evaluates whether a pragmatic 6-session-per-day schedule can be implemented in routine clinical practice and be associated with preliminary clinical improvement, while acknowledging that reduced treatment intensity may partly account for more modest response and remission rates.

### A pragmatic 5-day schedule may support routine clinical implementation despite moderate remission rates

The tolerability profile observed in this cohort is consistent with the broader accelerated rTMS literature rather than a novel safety signal. In our study, the implemented aiTBS protocol was well tolerated, and no serious AE were documented. A systematic review encompassing data from 43 trials (*n* = 881) revealed that accelerated rTMS protocols exhibited a comparable side effect profile to once-daily standard rTMS treatments.^18^ The 3 prevalent AE linked to aiTBS were headache (28.4%), fatigue (8.6%), and pain/discomfort at the stimulation site (8.3%), which are typically mild and resolve without intervention. The review also indicated that of 43 873 delivered aiTBS sessions, only 1 seizure was documented, yielding a per-session seizure rate of .0023%, which is analogous to the per-session seizure rate of .0017% found in once-daily conventional rTMS sessions.^25^

Prior research and established protocols guided the selection of stimulation parameters for the aiTBS protocol employed in our study. Our protocol retained standard iTBS session parameters (ie, 600 pulses per session) and an intended target stimulation intensity of 120% RMT. A tolerability-based titration range from 90% to 120% RMT was permitted during treatment initiation, allowing stimulation intensity to be temporarily adjusted according to individual tolerability. However, participant-level delivered intensity data were not available with sufficient granularity to determine the proportion of participants treated at each intensity level or to evaluate whether delivered stimulation intensity contributed to inter-individual differences in clinical outcomes or AE. We incorporated a 50-minute inter-stimulation interval, as animal studies have demonstrated that TBS is most effective in producing long-term potentiation with a minimum of approximately 1 hour between stimulation bouts.^26^ ^,^^27^ Overall, the protocol accelerated treatment delivery by administering 6 sessions per day, separated by 50-minute inter-stimulation intervals, over 5 consecutive days (ie, 30 total sessions and 18 000 iTBS pulses). These parameters provide cumulative pulse exposure similar to standard iTBS protocols while delivering treatment over substantially fewer days.

Compared with SNT’s 10-session daily schedule, this 6-session protocol may be easier to accommodate within routine clinical staffing and equipment constraints. Higher-intensity accelerated paradigms, such as SNT, may be difficult to reproduce with routine staffing, clinic hours, and equipment constraints.^15^ ^,^^20^ ^,^^21^ ^,^^28^ Thus, the contribution of the present study is that a 6-session-per-day, 5-day schedule retaining the cumulative pulse exposure of standard iTBS can be implemented within a routine tertiary clinical service. If confirmed in future randomized trials, this pragmatic schedule may represent a scalable compromise between treatment compression, cumulative stimulation exposure, potential antidepressant efficacy, and routine clinic deliverability.

### Limitations constrain causal inference but do not negate observed signal

Several limitations constrain interpretation of the present findings. First, the open-label, uncontrolled design limits causal inference. Without a sham-controlled or active comparator arm, observed symptom reductions cannot be definitively attributed to aiTBS, and expectancy effects may have contributed, particularly given the intensity and novelty of accelerated administration. Although symptom reductions were maintained at 2-month follow-up, spontaneous symptom fluctuation, regression to the mean, concurrent clinical care, and non-specific treatment effects cannot be excluded.

Second, baseline depressive symptom severity varied substantially. One participant had a baseline HAMD-17 score within the remission/minimal-symptom range, and nearly half of the cohort had mild HAMD-17 scores at treatment initiation. Because this naturalistic study relied on routine clinical assessment rather than a prespecified minimum baseline HAMD-17 threshold, some participants may have had lower symptom scores on the day of baseline rating despite meeting clinical criteria for a treatment-resistant major depressive episode. This variability should be considered when interpreting categorical response and remission rates.

Third, clinical heterogeneity may have influenced outcomes. Anxiety symptoms have been associated with a decreased likelihood of remission following rTMS.28 In addition, the inclusion of 3 participants with BP-II introduced diagnostic heterogeneity. Although sensitivity analysis excluding these participants showed that the primary HAMD-17 findings were not materially altered, the BP-II subgroup was too small to support conclusions regarding aiTBS for bipolar depression. Accordingly, the present findings should be interpreted primarily as reflecting outcomes among individuals with treatment-resistant MDD. Future studies should prospectively evaluate accelerated iTBS in adequately powered bipolar depression samples, with systematic monitoring for affective switching and mood stabilizer status.

Fourth, treatment-related confounding cannot be excluded. Concomitant benzodiazepine and anxiolytic exposure represent an important potential confounder. Although patients receiving benzodiazepines exceeding lorazepam 2 mg/day or equivalent, or anticonvulsant medications, were excluded, 28 participants (60.9%) had permitted benzodiazepine and/or other anxiolytic exposure during the aiTBS treatment course. Permitted benzodiazepines were prescribed on an as-needed basis, with instructions not to exceed lorazepam 2 mg per 24 hours or equivalent and not to use benzodiazepines after 5 PM on the evening preceding daily rTMS treatment. This exposure is clinically relevant because benzodiazepines modulate GABAergic inhibition and may influence cortical excitability and stimulation-induced plasticity.^23^ ^,^^29^ However, clinical studies examining benzodiazepine use during rTMS for depression have reported mixed findings.^30^ ^–^^32^ Medication-related confounding cannot be excluded because benzodiazepine/anxiolytic exposure was not available with sufficient detail regarding actual daily consumption, timing of use, medication type, dose, duration, frequency of as-needed use, or follow-up changes to support reliable differential-response analyses. Similarly, participant-level delivered stimulation intensity data were not available with sufficient granularity. Although the intended target intensity was 120% RMT and a tolerability-based titration range from 90% to 120% RMT was permitted during treatment initiation, we could not determine the proportion of participants treated at each intensity level or evaluate whether delivered intensity was associated with clinical response or adverse events.

Fifth, the modest sample size and single-center setting limit generalizability. The study was not powered to detect predictors of response, subgroup effects, or differences between diagnostic groups. Medication changes during follow-up were permitted and documented in clinical records but were not protocolized; therefore, 2-month outcomes may have been influenced by changes in psychotropic medications, psychotherapy engagement, or other concurrent clinical care after completion of the 5-day aiTBS course. In addition, the study was conducted at a single tertiary brain stimulation clinic, which may limit generalizability to other centers, service models, and patient populations. Finally, the study relied on clinical rating scales to assess depressive symptoms, anxiety symptoms, baseline global illness severity, and post-treatment global clinical change. Although these measures are clinically established, no validated biomarker of depression remission was available to corroborate symptom-based outcomes.

Overall, these limitations indicate that the findings should be interpreted as preliminary clinical outcome signals rather than definitive evidence of treatment efficacy. Adequately powered randomized controlled trials are needed to determine whether temporally compressed iTBS schedules produce clinical benefits beyond non-specific effects and routine clinical care.

### Future directions

First, randomized, sham-controlled trials directly comparing temporal compression to both standard once-daily iTBS and high-intensity accelerated paradigms are needed to disentangle the relative contributions of cumulative pulse load, targeting precision, and temporal architecture. Such trials should be powered to detect differences in remission rates and durability beyond 2 months.

Second, stratified designs examining diagnostic subtype are warranted. Future studies should either restrict inclusion to unipolar TRD or prospectively power bipolar depression subgroups with systematic monitoring for affective switching and mood stabilizer status.

Third, neurophysiological biomarkers, including TMS-EEG indices of cortical excitability, resting-state functional connectivity, or structural connectivity metrics, may clarify whether accelerated schedules produce distinct plasticity signatures relative to conventional protocols.

Fourth, moderator analyses examining comorbid anxiety, illness chronicity, baseline symptom severity, benzodiazepine/anxiolytic exposure, and medication burden may help identify subpopulations most likely to benefit from accelerated pulse exposure.

Finally, pragmatic multicenter trials are necessary to determine whether 5-day compressed schedules improve access, adherence, and health system efficiency relative to conventional treatment models.

## Conclusion

In this prospective, open-label, uncontrolled naturalistic cohort study, a 6-session-per-day aiTBS schedule delivering 18 000 total pulses over 5 days was feasible to implement, well-tolerated, and associated with sustained dimensional symptom reduction. However, in the absence of sham or active comparator condition, these findings should be interpreted as preliminary clinical outcome signals rather than evidence of antidepressant efficacy.

The present findings support continued investigation of temporally compressed iTBS as a potentially scalable neuromodulation strategy while underscoring the need for randomized controlled trials to clarify the relative contributions of dose intensity, targeting precision, and session architecture.

## Supporting information

10.1017/S1092852926101205.sm001 Khanna et al. supplementary material Khanna et al. supplementary material

## Data Availability

The data that support the findings of this study are available from the corresponding author upon reasonable request.

## Supplementary material

The supplementary material for this article can be found at https://doi.org/10.1017/S1092852926101205.

## Long descriptions

### Figure 1. Long description

The flowchart is divided into three vertical sections.

1. ELIGIBILITY section at the top.

- Referred for aiTBS by psychiatrist (n = 75).

- An arrow points right to Excluded by research staff (n = 15), with sub-reasons: Unreachable over the phone (n = 13) and Declined treatment (n = 2).

- The main flow continues down to Participants reached by research staff and consented (n = 60).

- An arrow points right to Excluded before treatment initiation (n = 1) due to Withdrew consent (n = 1).

2. TREATMENT section in the middle.

- The main flow continues down to Attended treatment initiation (n = 59).

- An arrow points right to Discontinued treatment (n = 2), with sub-reasons: Pain (n = 1) and Nausea (n = 1).

- The main flow continues down to Completed treatment (n = 57).

- An arrow points right to Excluded from analysis (n = 11) due to Lost to post-treatment and or follow-up (n = 12).

3. INCLUDED section at the bottom.

- The final box in the main vertical flow shows Studies included in review (n = 46).

Navigate back to Figure 1..

### Table 1. Long description

The table is organized into two columns: Variable and Value.

* Age: mean 44.33 with a range of 21 to 77.

* Sex: 25 males (54.4 percent) and 21 females (45.7 percent).

* Diagnosis: 43 participants (93.5 percent) with Major Depressive Disorder and 3 (6.5 percent) with Bipolar Disorder-II.

* Prior and concurrent treatments: 25 (54 percent) used Atypical antipsychotics; 28 (61 percent) used Benzodiazepines or other anxiolytics; 33 (71 percent) underwent Psychotherapy; 11 (24 percent) underwent Electroconvulsive therapy.

* Baseline symptom severity (mean and S D):

- CGI-S: 4.35 (0.67)

- HAMD-17: 17.59 (5.69)

- GAD-7: 10.76 (5.50)

- PHQ-9: 18.63 (5.16)

Abbreviations include CGI-S (Clinical Global Impression-Severity scale), GAD-7 (General Anxiety Disorder scale-7), HAMD-17 (Hamilton Depression Rating Scale-17), and PHQ-9 (Patient Health Questionnaire-9).

Navigate back to Table 1..

### Figure 2. Long description

The schematic is divided into two horizontal panels.

Panel A. Distributed Once-Daily iTBS Schedule.

A horizontal timeline shows a sequence of orange vertical bars representing pulse trains. One train consists of 10 clusters delivered over 2 seconds. An 8 second interval separates each train. A total of 20 trains are delivered once daily. A circular inset zooms into a single cluster, showing 9 pulses at 50 Hertz with a 200 millisecond interval between bursts.

Panel B. Temporally Compressed Dose-Equivalent aiTB S.

The timeline shows 1 session consisting of 20 trains delivered over approximately 3 minutes. Each 10 second block includes a 2 second train and an 8 second inter-train interval. A 50 minute interval separates these sessions. The timeline concludes with a total of 6 sessions, or 120 trains, per day. A circular inset zooms into a single train, showing 30 pulses at 50 Hertz.

Both panels use a blue horizontal arrow to indicate the progression of time from left to right.

Navigate back to Figure 2..

### Table 2. Long description

The table presents data for 46 participants.

1. HAMD-17: Mean scores decreased from 17.6 at baseline to 12.1 post-treatment and 12.0 at follow-up. Post-treatment, 28.3 percent met clinically meaningful improvement and 26.1 percent reached remission. At follow-up, 37.0 percent met improvement criteria and 32.6 percent reached remission.

2. PHQ-9: Mean scores decreased from 18.6 at baseline to 13.8 post-treatment and 13.4 at follow-up. Post-treatment, 80.4 percent improved and 2.2 percent reached remission. At follow-up, 78.3 percent improved and 6.5 percent reached remission.

3. GAD-7: Mean scores decreased from 10.8 at baseline to 8.3 post-treatment and 8.3 at follow-up. Post-treatment, 60.9 percent improved and 28.3 percent reached remission. At follow-up, 65.2 percent improved and 32.6 percent reached remission.

4. CGI-I: Mean scores decreased from 4.3 at baseline to 3.2 post-treatment and 3.0 at follow-up. Post-treatment, 52.2 percent improved and 8.7 percent reached remission. At follow-up, 60.9 percent improved and 6.5 percent reached remission.

Statistically significant differences compared to baseline are noted for all post-treatment and follow-up mean scores. Higher scores denote worse symptoms across all scales.

Navigate back to Table 2..

### Figure 3. Long description

A bar graph with the y-axis labeled Scores from 0 to 25 and the x-axis labeled Clinical Assessments. The data is grouped into four assessment types, each shown at Pre, Post, and Follow up intervals.

* HAMD-17. Solid dark gray bars. Pre is approximately 17.5. Post is approximately 12 with an asterisk. Follow up is approximately 12 with an asterisk.

* CGI. White bars with blue outlines. Pre is approximately 4.5. Post is approximately 3.5 with an asterisk. Follow up is approximately 3 with an asterisk.

* GAD-7. White bars with horizontal blue stripes. Pre is approximately 11. Post is approximately 8.5 with an asterisk. Follow up is approximately 8.5 with an asterisk.

* PHQ-9. White bars with diagonal blue stripes. Pre is approximately 18.5. Post is approximately 14 with an asterisk. Follow up is approximately 13.5 with an asterisk.

All bars include vertical error bars representing standard deviation. Asterisks indicate statistically significant improvement relative to baseline.

Navigate back to Figure 3..
