# Supplementary material for: Clinical outcomes following five-day accelerated intermittent theta burst stimulation for treatment-resistant depression in routine clinical practice: A prospective naturalistic cohort study
Source: CNS Spectr. 2026 Oct 15;31(1):e43. doi: 10.1017/S1092852926101205 (PMC13732625; doi:10.1017/S1092852926101205)
Supplement: Supplementary file 1 [file S1092852926101205sup001.docx]

**Clinical Outcomes Following Five-Day Accelerated Intermittent Theta Burst Stimulation for Treatment-Resistant Depression in Routine Clinical Practice: A Prospective Naturalistic Cohort Study**

Asmi Khanna^1,2^, Adrian I. Espiritu^2,3,4,5^, Lynn Zhu^2,6^, Mervin Blair^7^, Olivia Giovannetti^2^, Amanda Abate^2^, Janice Knight^2^, Luisa Deutsch^2^, Robyn Waxman^2,3^, Amer M. Burhan^2,3,*^

^1^Department of Human Biology, Faculty of Arts & Science, University of Toronto, Toronto, Ontario, Canada

^2^Ontario Shores Centre for Mental Health Sciences, Whitby, Ontario, Canada

^3^Department of Psychiatry, Temerty Faculty of Medicine, University of Toronto, Toronto, Ontario, Canada

^4^Department of Medicine, Division of Neurology, University of Toronto, Toronto, Ontario, Canada

^5^Department of Clinical Epidemiology, College of Medicine, University of the Philippines Manila, Manila, Philippines

^6^Faculty of Health Sciences, Ontario Tech University, Oshawa, Canada

^7^Lawson Research Institute, London ON, Canada

***Corresponding author contact information:**

Amer M. Burhan, MBCHB MSc FRCPC

[burhana@ontarioshores.ca](mailto:burhana@ontarioshores.ca)

(905) 430-4055 Ext. 4019

Ontario Shores Centre for Mental Health Sciences

700 Gordon Street, Whitby, ON, L1N 0H7

**Authors’ emails:**

Asmi Khanna, HBSc Candidate

[khannaa@ontarioshores.ca](mailto:khannaa@ontarioshores.ca)

Adrian I. Espiritu, MD FPNA

[espiritua@ontarioshores.ca](mailto:espiritua@ontarioshores.ca)

Lynn Zhu, PhD

[zhuly@ontarioshores.ca](mailto:zhuly@ontarioshores.ca)

Mervin Blair, PhD CPSYCH

[blairmer@ontarioshores.ca](mailto:blairmer@ontarioshores.ca)

Olivia Giovannetti, PhD MSC

[giovannettio@ontarioshores.ca](mailto:giovannettio@ontarioshores.ca)

Amanda Abate MD FRCPC

[abatea@ontarioshores.ca](mailto:abatea@ontarioshores.ca)

Janice Knight

[knightj@ontarioshores.ca](mailto:knightj@ontarioshores.ca)

Luisa Deutsch MD MSc

[deutschl@ontarioshores.ca](mailto:deutschl@ontarioshores.ca)

Robyn Waxman, MD FRCPC

[waxmanro@ontarioshores.ca](mailto:waxmanro@ontarioshores.ca)

Table of Contents

[Supplementary Table 1. STROBE Checklist	4](#_Toc223129587)

[Supplementary Statistical Analysis Overview	8](#_Toc223129588)

## **Supplementary Table 1.** STROBE Checklist. Strengthening the reporting of observational studies in epidemiology (STROBE) Checklist

|  | Item No. | Recommendation | Page  No. |
| --- | --- | --- | --- |
| **Title and abstract** | 1 | (*a*) Indicate the study’s design with a commonly used term in the title or the abstract | 1 |
|  |  | (*b*) Provide in the abstract an informative and balanced summary of what was done and what was found | 3 |
| Introduction | | | |
| Background/rationale | 2 | Explain the scientific background and rationale for the investigation being reported | 1 |
| Objectives | 3 | State specific objectives, including any prespecified hypotheses | 2 |
| Methods | | | |
| Study design | 4 | Present key elements of study design early in the paper | 2 |
| Setting | 5 | Describe the setting, locations, and relevant dates, including periods of recruitment, exposure, follow-up, and data collection | 2 |
| Participants | 6 | (*a*) *Cohort study*—Give the eligibility criteria, and the sources and methods of selection of participants. Describe methods of follow-up  *Case-control study*—Give the eligibility criteria, and the sources and methods of case ascertainment and control selection. Give the rationale for the choice of cases and controls  *Cross-sectional study*—Give the eligibility criteria, and the sources and methods of selection of participants | 2-3 |
|  |  | (*b*) *Cohort study*—For matched studies, give matching criteria and number of exposed and unexposed  *Case-control study*—For matched studies, give matching criteria and the number of controls per case | N/A |
| Variables | 7 | Clearly define all outcomes, exposures, predictors, potential confounders, and effect modifiers. Give diagnostic criteria, if applicable | 3 |
| Data sources/ measurement | 8* | For each variable of interest, give sources of data and details of methods of assessment (measurement). Describe comparability of assessment methods if there is more than one group | 4 |
| Bias | 9 | Describe any efforts to address potential sources of bias | 4-5 |
| Study size | 10 | Explain how the study size was arrived at | 5 |
| Quantitative variables | 11 | Explain how quantitative variables were handled in the analyses. If applicable, describe which groupings were chosen and why | 5 |
| Statistical methods | 12 | (*a*) Describe all statistical methods, including those used to control for confounding | 5 |
|  |  | (*b*) Describe any methods used to examine subgroups and interactions | 5 |
|  |  | (*c*) Explain how missing data were addressed | 5 |
|  |  | (*d*) *Cohort study*—If applicable, explain how loss to follow-up was addressed  *Case-control study*—If applicable, explain how matching of cases and controls was addressed  *Cross-sectional study*—If applicable, describe analytical methods taking account of sampling strategy | 5 |
|  |  | (*e*) Describe any sensitivity analyses | N/A |
| Participants | 13* | (a) Report numbers of individuals at each stage of study—eg numbers potentially eligible, examined for eligibility, confirmed eligible, included in the study, completing follow-up, and analysed | 6 |
|  |  | (b) Give reasons for non-participation at each stage | 6 |
|  |  | (c) Consider use of a flow diagram | Figure 1 |
| Descriptive data | 14* | (a) Give characteristics of study participants (eg demographic, clinical, social) and information on exposures and potential confounders | 6 |
|  |  | (b) Indicate number of participants with missing data for each variable of interest | 6 |
|  |  | (c) *Cohort study*—Summarise follow-up time (eg, average and total amount) | 6 |
| Outcome data | 15* | *Cohort study*—Report numbers of outcome events or summary measures over time | 7 |
|  |  | *Case-control study—*Report numbers in each exposure category, or summary measures of exposure | N/A |
|  |  | *Cross-sectional study—*Report numbers of outcome events or summary measures | N/A |
| Main results | 16 | (*a*) Give unadjusted estimates and, if applicable, confounder-adjusted estimates and their precision (eg, 95% confidence interval). Make clear which confounders were adjusted for and why they were included | 6-7 |
|  |  | (*b*) Report category boundaries when continuous variables were categorized | 6-7 |
|  |  | (*c*) If relevant, consider translating estimates of relative risk into absolute risk for a meaningful time period | N/A |
| Other analyses | 17 | Report other analyses done—eg analyses of subgroups and interactions, and sensitivity analyses | N/A |
| Key results | 18 | Summarise key results with reference to study objectives | 7 |
| Limitations | 19 | Discuss limitations of the study, taking into account sources of potential bias or imprecision. Discuss both direction and magnitude of any potential bias | 11 |
| Interpretation | 20 | Give a cautious overall interpretation of results considering objectives, limitations, multiplicity of analyses, results from similar studies, and other relevant evidence | 8-10 |
| Generalisability | 21 | Discuss the generalisability (external validity) of the study results | 11 |
| Other information | | | |
| Funding | 22 | Give the source of funding and the role of the funders for the present study and, if applicable, for the original study on which the present article is based | 16 |

## **Supplementary Statistical Analysis Overview.** All statistical analyses were conducted using R (RStudio, version 2026.01.1+403). Longitudinal symptom trajectories were analyzed using linear mixed-effects models to account for repeated measures within participants across time.

**Software And Packages**

Analyses were performed using the following R packages:

- lme4 (version 4_1.1-38) for model estimation
- lmerTest (version 3.2-0) for inference and Satterthwaite degrees of freedom approximation
- emmeans (version 2.0.1) for estimated marginal means and post hoc comparisons
- effectsize (version 1.0.1), if applicable, for standardized effect size estimation

**Model Specification**

For each continuous outcome (HAMD-17, PHQ-9, and GAD-7), a separate linear mixed-effects model was fitted using restricted maximum likelihood (REML) estimation.

*The general model structure was:*

Outcome ~ Time + (1 | Participant)

*Where:*

- Time was modeled as a categorical fixed effect (baseline, post-treatment, two-month follow-up)
- Participant was included as a random intercept to account for within-subject correlation of repeated measures

Degrees of freedom were estimated using the Satterthwaite approximation.

**Post Hoc Comparisons**

When the overall effect of Time was statistically significant, pairwise comparisons between time points were performed using estimated marginal means with Bonferroni correction for multiple comparisons (adjusted significance threshold p < 0.017 for three comparisons).

**Effect Size Estimation**

Standardized within-subject effect sizes (Cohen’s d) were calculated using model-estimated marginal means and pooled baseline standard deviation. Ninety-five percent confidence intervals were derived from model-based estimates.

Effect sizes were interpreted according to conventional benchmarks:

- Small: d ≈ 0.2
- Medium: d ≈ 0.5
- Large: d ≥ 0.8

**Handling of Missing Data**

The mixed-effects modeling framework allows inclusion of participants with incomplete follow-up data without requiring imputation. All available observations were included in analyses under the assumption that data were missing at random.

**Categorical Outcomes**

Categorical outcomes were calculated descriptively according to predefined thresholds. HAMD-17 response was defined as ≥50% reduction from baseline, and HAMD-17 remission was defined as HAMD-17 ≤7. PHQ-9 and GAD-7 improvement categories in Table 2 were defined descriptively as any reduction from baseline, while remission was defined as PHQ-9 <5 and GAD-7 ≤4. CGI-I improvement ratings were summarized descriptively at post-treatment and follow-up and were not used to define remission. Categorical outcomes were not subjected to inferential hypothesis testing.

CGI-S was summarized as a baseline measure of clinician-rated global illness severity. CGI-I was summarized descriptively at post-treatment and follow-up. Because CGI-S and CGI-I assess different constructs, CGI was not modeled as a continuous longitudinal outcome.

**Example Model Syntax (Hamd-17)**

```r

library(lme4)

library(lmerTest)

library(emmeans)

model_hamd <- lmer(HAMD17 ~ Time + (1 | Participant), data = dataset, REML = TRUE)

anova(model_hamd)

emmeans(model_hamd, pairwise ~ Time, adjust = "bonferroni")

```
